# A Case of Candida auris Candidemia in an Immunocompetent Traumatic Brain Injury Patient Post Ventriculoperitoneal Shunt and Peripherally Inserted Central Catheter Line

**DOI:** 10.7759/cureus.8850

**Published:** 2020-06-26

**Authors:** Elvina Lingas, Maria del Mar Lucio Paredes, Mansura Jahan, Parinitha Bathina, Michelle Dahdouh

**Affiliations:** 1 Internal Medicine, St. Barnabas Hospital, Bronx, USA; 2 Internal Medicine, St. Barnabas Hospital Health System, Bronx, USA; 3 Infectious Disease, St. Barnabas Hospital Health System, Bronx, USA

**Keywords:** candida auris, healthcare associated infection, total perenteral nutrition

## Abstract

Among the candida species, Candida auris (C. auris) is multidrug-resistant and is associated with invasive hospital-acquired infection and high mortality. We are going to present a case report about C. auris in an immunocompetent patient. The patient was admitted due to traumatic sub dural hemorrhage and during the hospital course he developed fever. Blood cultures grew C. auris. Our aim is to raise awareness regarding C. auris for early detection and prevention of hospital-acquired transmission of the infection.

## Introduction

*Candida auris* (*C. auris*) is a rapidly emerging hospital-acquired and multidrug-resistant fungal pathogen that is associated with high morbidity and mortality. In the report released by the Centers for Disease Control (CDC) on December 31, 2019, this pathogen had been detected in at least 16 states, i.e., New York is the one with the maximum number of cases (461 out of 988) [1]. Early diagnosis of *C. auris* is imperative for effective treatment and control of infection. For purposes of early identification, we will present a patient who was initially admitted due to traumatic brain injury (TBI) requiring placement of ventriculoperitoneal (VP) shunt and peripherally inserted central catheter (PICC) line for total parenteral nutrition (TPN), with his hospital course complicated by *C. auris* candidemia and Pseudomonas VP shunt infection.

## Case presentation

**Figure 1:**
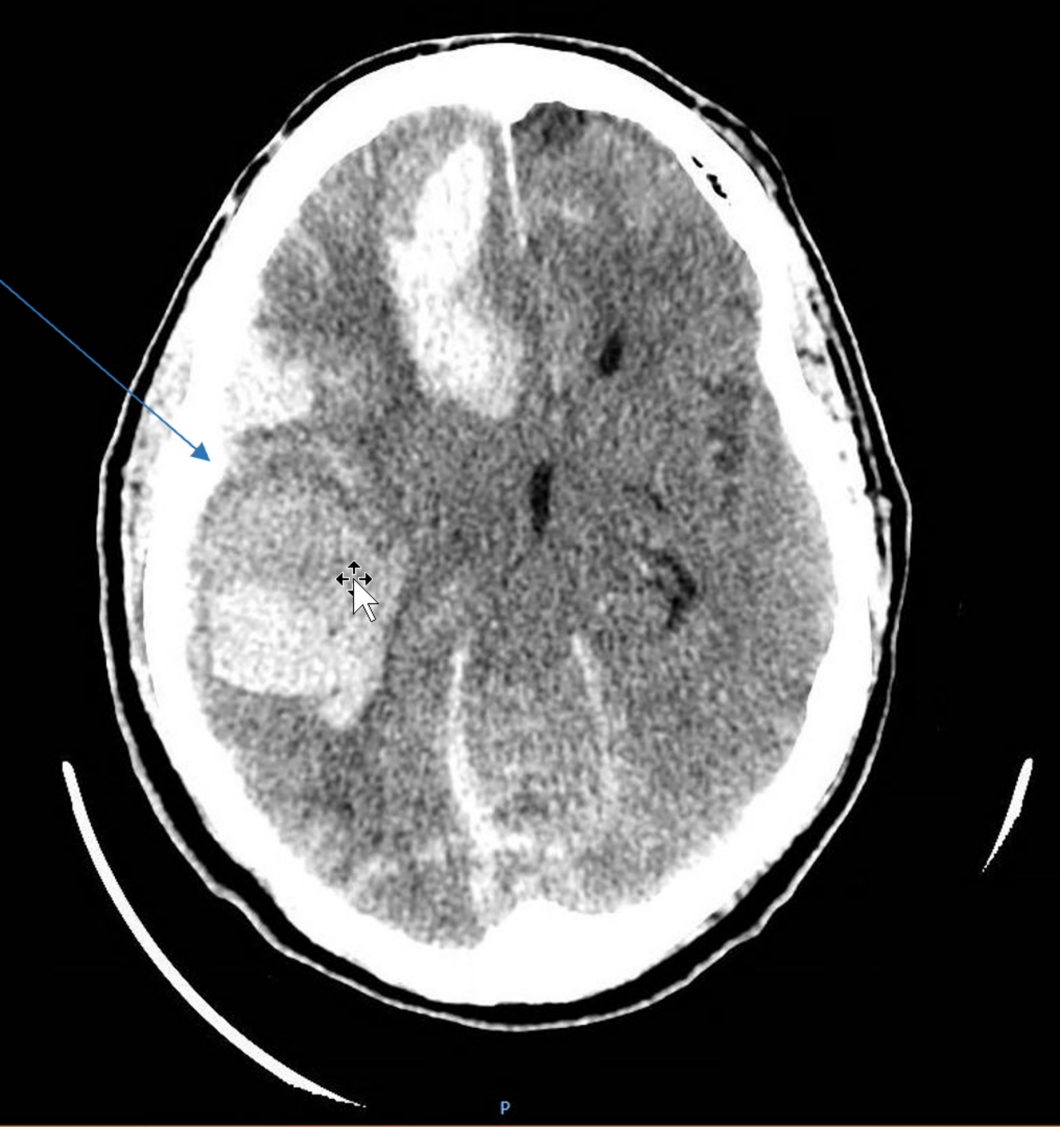
Extensive left frontotemporal and parenchymal hematomas, intraventricular hemorrhage, and subarachnoid and subdural hemorrhage.

**Figure 2:**
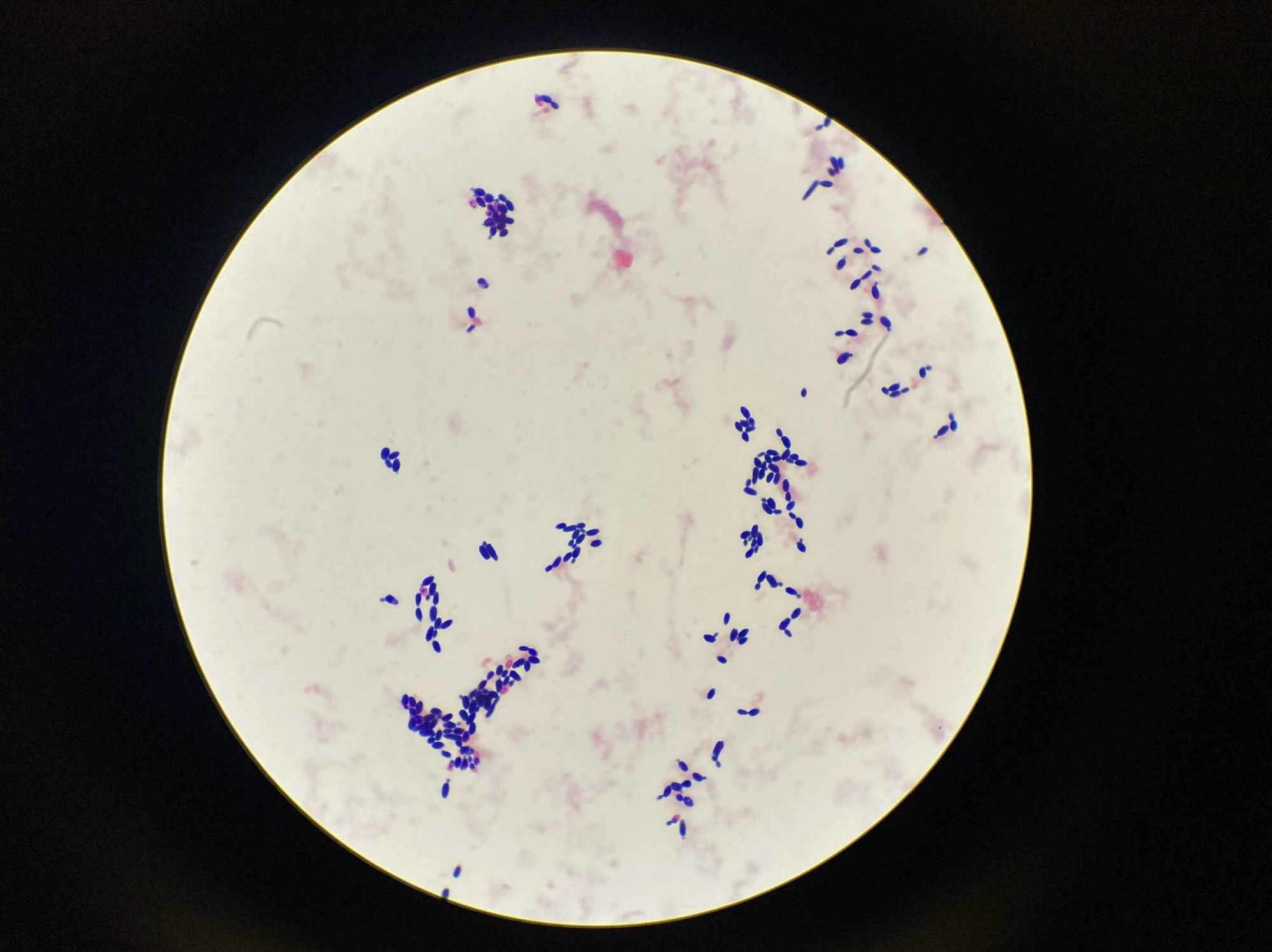
C. auris isolated from blood culture.

A 61-year-old male, born in the United States, with no prior medical history, was brought in by EMS to the ED for trauma due to mechanical fall. The CT brain demonstrates right-sided subdural and epidural bleeding without any evidence of midline shift. The CT brain is shown in Figure 1. The patient was initially admitted for TBI with a worsening mental status. The patient had to be intubated to protect the airway. During his prolonged stay in the surgical intensive care unit (SICU) the patient had difficulty being weaned off from mechanical ventilator and subsequently required tracheostomy placement. Due to traumatic hydrocephalus, the patient also required a VP shunt placed as well. A PICC line was also placed by the surgery team to start TPN as the patient's position had to be maintained 10 degrees as per neurosurgery and tube feeding could not be started at that time. The patient was stable, without any infection wise until nine days into the hospitalization when he developed worsening fevers, with the highest of 101.7 degrees Fahrenheit (normal range: 97.7-99.5 degrees Fahrenheit). At that time there was a concern for VP shunt site infection with cerebrospinal fluid (CSF) leak and worsening right subdural hematoma. Different cultures including respiratory, urine, blood, and CSF cultures were obtained from the shunt. Broad-spectrum antibiotics namely IV vancomycin and meropenem were started. The PICC line was discontinued on the same day. The CSF culture eventually grew *Pseudomonas aeruginosa* that was pan sensitive and blood culture was growing candida. A slide showing *C. auris* is shown in Figure 2. Micafungin was added to the treatment regimen, the candida was identified as auris, contact isolation was initiated, and micafungin was switched to amphotericin as the patient was still febrile. Repeat blood cultures were obtained, echocardiography was ordered to rule out endocarditis, and ophthalmology was called to perform an eye exam to rule out fungal endophthalmitis. Initial transthoracic echocardiogram (TTE) showed possible vegetation; however, transesophageal echocardiography (TEE) ruled out endocarditis. Regarding Pseudomonas in the CSF, neurosurgery was not able to externalize and drain CSF until culture negative and put a new shunt when culture negative as the patient's hydrocephalus was quite severe. Antimicrobial therapy was switched to cefepime for six weeks due to lack of removal of VP shunt and *C. auris* was found to be sensitive to micafungin (minimum inhibitory concentration, MIC < 4) and the patient in total received 14 days of both amphotericin B and micafungin. The fever curve improved and the patient was then discharged to a nursing home for further physical therapy and allowed time for recovery. 

## Discussion

Candidemia is defined as the presence of any type of Candida species in a blood culture. This should never be considered a contaminant. It is important to discuss this subject, with the increasing prevalence of the non-albicans Candida species infection-associated morbidity, especially in critically ill patients in an inpatient setting. A multicenter surveillance study was conducted in the United States between 2004 and 2008 which showed that 54% of the bloodstream isolates in 2019 represented non-albicans Candida species, 46% of blood isolates represented *C. albicans*, while 26% of all cases of candidemia was represented by *C. glabrata* which is followed by *C. parapsilosis* (16%), *C. tropicalis* (8%), and *C. krusei* (3%) [2]. The worrisome fact is that some species of non-albicans Candida are resistant to fluconazole. 

In 2016, the United States CDC issued warnings about the existence of a multidrug-resistant Candida species, known as *C. auris* [3]. It is observed that this pathogen has been implicated as one of the causative agents of invasive healthcare-associated infection with high mortality rates. In 2009, *C. auris* was first described in Japan, upon retrospective testing of isolates, the earliest known infections occurred in 1996 in South Korea. *C. auris* has been detected in more than 30 countries and has caused outbreaks in healthcare facilities [2]. With more recent studies and understanding of the pathogenesis of Candida, it is now known that the major routes of bloodstream infection are through the gastrointestinal (GI) tract and IV catheters (especially TPN). It is observed that *C. auris* has been mostly detected in patients with extensive hospitalizations including acute healthcare facilities and nursing homes which have some type of invasive devices like central lines, PICC lines, cholecystostomy tubes, Foley catheters, and others [2, 4].

Blood cultures taken promptly, in patients who are suspected to have developed fungemia is the best diagnostic tool (despite the low sensibility of this test). About 50% of the cultures obtained have been negative for invasive candidiasis. There are data and research studies suggestive of nonculture tests that are more reliable in diagnosing both candidemia and deep-seated candidiasis; some of them are mannan or anti mannan, *C. albicans* germ tube antibody, 1,3-β-d-glucan, polymerase chain reaction (PCR), and T2Candida panel [5]. To efficiently utilize the available testing tools the clinician needs to understand the pretest likelihood of the invasive candidiasis and the test performance for the most common disease manifestation in a given patient [2]. Considering the enormous burden that this pathogen can put on a healthcare facility and its patients, prevention is one of the most important actions to be taken when attempting eradication and before achieving the same. The CDC recommends strict contact precautions when dealing with a patient from any healthcare facility or nursing home that has a previous history of *C. auris* colonization. These contact precautions include proper hand hygiene, cohorting patients with a similar history, and implementing regular environmental disinfection. *C. auris* has been cultured from the surfaces of multiple locations in patient’s rooms; they have been observed to persist in sites including high-touch surfaces like bedside tables, bed rails as well as in general environmental surfaces away from the patient, such as windowsills. *C. auris* has also been identified on portable equipment that is shared between patients like glucometers, temperature probes, blood pressure cuffs, ultrasound machines, nursing carts, and crash carts [3].

It is important to follow all manufacturer’s directions while using surface disinfectants; for example, applying the product for the correct contact time. Some products with *C. albicans* or fungicidal claims may not be effective against *C. auris*, products solely dependent on quaternary ammonia compounds (QACs) are NOT effective against *C. auris* as evidenced by accumulating data [3]. There is a high possibility of false negatives when performing repetitive swabs in patients requiring complex medical care, like continuous ventilator support and other invasive procedures, thereby CDC recommends against reassessment of colonization in such patients. Reassessment should be only considered in settings where patients have had significant improvement, and it has been more than three months since the last positive test of *C. auris*. The reassessments should at a minimum involve testing of swabs from the axillary region, groin area, and sites that yielded *C. auris* on previous specimens (e.g., urine and sputum). To have a reliable result, the patient must not be receiving antifungal medications active against *C. auris* at the time of these assessments. It is reasonable to wait one week between the last receipt of antifungal medication and testing for *C. auris* colonization but the optimal time to perform the test has not yet been established. In cases where topical antiseptic (e.g., chlorhexidine) or similar products are being used, it is important to wait at least 48 hours before testing for *C. auris* colonization be performed. The CDC recommends for infection control precautions specific for *C. auris* to be discontinued only if a patient or resident has two negative colonization tests at least one week apart in situations where reassessment is considered appropriate [3]. 

The choice of treatment for candidemia is primarily echinocandins and azoles. Amphotericin B is only a second-line agent secondary to its multiple side effects. It is important to understand that the removal of the invasive device alone is not enough, we can efficiently cut down the high mortality rate by the initiation of appropriate antifungal medication on time. However, due to multiple reports of multidrug resistance-related to *C. auris*, the recommendation is to start echinocandins when this pathogen is detected [6]. The patient should be monitored with daily cultures. If patients are repeatedly positive in cultures or there is no clinical improvement in the patient’s condition, management should be changed to amphotericin B. Unfortunately, there is a lack of research and evidence in multiple aspects of treatment and management of candidemia, like combination therapy and duration of treatment. At this time, combination therapy is not recommended. Duration of treatment has been established for up to two weeks after blood cultures have turned up negative. 

Reviewing the data available so far and observing the high mortality rate of untreated candidemia (which can reach 60%), we want to emphasize that healthcare professionals should be aware and must consider the possibility of candidemia in patients with prolonged ventilator support and requiring invasive devices like central lines and TPN. We want to strongly insist that identifying high-risk patients, and intervening early with adequate and prompt medical management would make a great difference and lower the mortality associated with the infection. 

## Conclusions

Aggressive infection control measures including using selective antibiotics, antifungals, hand hygiene, and contact precautions are imperative to reduce the spread of *C. auris*. It is important to diagnose and treat as early as possible to improve morbidity, mortality, and prevent transmission. Patients who are at high risk, including devices that have been placed for long periods of time, should be tested for *C. auris* and treated early.
